# Methionine restriction improves lipid metabolism disorders and enhances immunity in obese mice through liver lipid remodeling

**DOI:** 10.3389/fnut.2026.1837878

**Published:** 2026-06-26

**Authors:** Tianxin Ma, Sen Yang, Xing Xie, Xinjing Zhang, Cunqi Ye, Lu Zhang, Zongcai Tu

**Affiliations:** 1 College of Chemistry and Materials, Jiangxi Normal University, Nanchang, Jiangxi, China; 2 Institute of Immunology and Bone Marrow Transplantation Center, The First Affiliated Hospital, School of Medicine, Zhejiang University, Hangzhou, China; 3 Liangzhu Laboratory, Zhejiang University Medical Center, Hangzhou, China; 4 College of Life Sciences, Jiangxi Normal University, Nanchang, Jiangxi, China; 5 State Key Laboratory of Food Science and Resources, Nanchang University, Nanchang, Jiangxi, China; 6 Zhejiang Provincial Key Laboratory for Cancer Molecular Cell Biology, Life Sciences Institute, Zhejiang University, Hangzhou, China; 7 College of Pharmacy, Jiangxi Normal University, Nanchang, Jiangxi, China

**Keywords:** anti-inflammatory, bacterial infection resistance, lipid remodeling, methionine restriction diet, weight loss

## Abstract

**Background:**

Methionine-restriction diet (MRD) has been reported to improve obesity-related metabolic disorders, but its effects during weight loss and the underlying mechanisms remain unclear. This study investigated whether reducing dietary methionine could improve metabolic status, reshape lipid composition, and influence inflammatory and antibacterial responses in high-fat diet (HFD) induced obese mice.

**Methods:**

Male C57BL/6 mice were fed a high-fat diet for 16 weeks to induce obesity and were then assigned to continued HFD, a normal control diet (NC), or a MRD for 21 days. Body weight, food and water intake, tissue mass, and serum biochemical indices were measured. Targeted metabolomics and lipidomics of liver and serum were performed by liquid chromatography-mass spectrometry. Serum inflammatory factors were determined by enzyme-linked immunosorbent assay, and resistance to bacterial infection was evaluated using a *Listeria monocytogenes* challenge model.

**Results:**

Both the NC and MRD reduced body weight and epididymal fat mass compared with the continuous HFD, whereas routine serum biochemical indices showed no significant differences between the two diet-switch groups. Targeted metabolomics revealed only limited differences between the MRD and NC groups in both liver and serum, suggesting that MRD did not induce broad metabolic disturbance during weight loss. In contrast, lipidomics showed clear lipid remodeling, especially in the liver, characterized by increased phosphatidylethanolamine and phosphatidylserine and decreased phosphatidic acid, lysophosphatidylcholine, and diacylglycerol, while total phosphatidylcholine remained largely unchanged. MRD also reduced the average acyl-chain length and saturation of hepatic phosphatidylethanolamine and phosphatidylcholine. Functionally, MRD reduced IL-1β and IL-6 compared with NC, whereas TNF-α was reduced in both diet-switch groups compared with HFD., as well as reduced bacterial burdens in the liver and spleen after *Listeria monocytogenes* infection.

**Conclusion:**

These findings indicate that MRD during weight loss does not broadly disrupt systemic metabolism, but is associated with selective hepatic lipid remodeling and improved inflammatory and antibacterial responses. This study extends current understanding of dietary MRD by linking weight-loss intervention with liver lipid reorganization and host defense, and provides experimental support for nutritional strategies targeting obesity-related metabolic dysfunction.

## 1 Introduction

The global obesity epidemic is expanding rapidly, affecting both developed and developing countries. Rising obesity rates are associated with heightened health risks and a substantial economic burden (1). The underlying cause of obesity is an imbalance between energy intake and expenditure, characterizing by excessive adipose tissue accumulation. Obesity is a major risk factor for numerous metabolic diseases, including fatty liver disease, type 2 diabetes mellitus, and coronary artery disease, as well as certain cancer. Moreover, obesity compromises immune function and diminishes resistance to pathogens, as exemplified by the increased mortality observed among obese patients during the COVID-19 pandemic (2). Consequently, weight loss represents a cornerstone strategy for mitigating obesity-related complications. Common approaches for weight loss include anti-obesity medications, bariatric surgery, and lifestyle interventions (3). Among these, dietary interventions are the most prevalent. An optimal dietary strategy for obesity management should be safe, effective, health-promoting, economically feasible, and culturally adaptable. Such regimens include low-fat, low-calorie, low-carbohydrate, and high-protein diets, along with formulated diets, the Mediterranean diet, and intermittent fasting (4).

Methionine is an essential sulfur-containing amino acid involved in protein synthesis, one-carbon metabolism, methylation reactions, redox regulation, and lipid metabolism (5). Dietary methionine is obtained from various sources, which can be broadly categorized into two groups: animal-based foods such as lean meat, eggs, milk, and fish, and plant-based foods including soybeans, nuts, quinoa, and whole wheat (6). After cellular uptake, methionine is converted by methionine adenosyltransferase (MAT) into S-adenosylmethionine (SAM), the major cellular methyl donor. SAM-dependent methylation reactions regulate DNA, RNA, histones, proteins, phospholipids, and metabolites, thereby linking methionine availability to epigenetic regulation, gene expression, membrane lipid metabolism, and cellular homeostasis. After methyl donation, SAM is converted to S-adenosylhomocysteine (SAH) and subsequently to homocysteine, which can either be remethylated to regenerate methionine or enter the transsulfuration pathway. Thus, methionine metabolism constitutes a central metabolic network connecting methyl-donor balance, redox homeostasis, and lipid metabolic regulation (7). This methionine–SAM axis is particularly relevant to hepatic lipid metabolism through the phosphatidylethanolamine N-methyltransferase (PEMT) pathway, in which SAM is used as the methyl donor to catalyze the sequential methylation of phosphatidylethanolamine (PE) to phosphatidylcholine (PC), thereby directly linking methionine availability to phospholipid remodeling and membrane lipid homeostasis. PE and PC are the two most abundant phospholipids in cell membranes and intracellular lipid structures, collectively accounting for 60–80% of total phospholipids and playing central roles in membrane integrity, organelle function, lipid transport, inflammatory regulation, and lipid homeostasis (8). Notably, both clinical and animal studies indicate that obesity is commonly associated with elevated triglyceride levels and reduced PE concentrations (9). Although weight loss improved obesity-associated lipid abnormalities, the serum lipidome did not fully normalize. Fatty acyls and glycerolipids, which were elevated in severe obesity, tended to decrease after weight loss, whereas reduced glycerophospholipids and sterol lipids showed only partial recovery. Persistent alterations in PCs, ether-linked PCs, LPEs, sphingomyelins, and inflammation-related lipids such as adrenic acid and 12-HETE suggest ongoing phospholipid remodeling defects, delayed sphingolipid normalization, and residual lipid-mediated inflammation during weight loss (10). These findings suggest that lipid metabolic dysregulation may persist during weight loss. Therefore, it is of great significance to improve lipid metabolism disorders during the fat loss process.

The dietary level of methionine profoundly influences cellular and systemic metabolism. Methionine restriction (MR), usually achieved using a methionine-restricted diet (MRD), has been shown to extend lifespan in yeast (11), *Drosophila melanogaster* (12), and mice (13, 14), and to improve metabolic phenotypes in obesity (15, 16), liver dysfunction (13, 17), oxidative stress, insulin resistance, and diabetes (18). In classical rodent MR models, dietary methionine is commonly reduced from 0.86% to 0.17%, corresponding to an approximately 80% reduction in methionine intake. This concentration was selected because it falls within the effective range reported to induce MR-related metabolic responses, while remaining above the threshold associated with severe methionine deprivation (19). Mechanistically, these effects have been associated with conserved nutrient-sensing and stress-adaptive pathways, including TOR/mTORC1 signaling, eIF2α-ATF4–FGF21 signaling, mitochondrial adaptation, redox regulation, inflammatory modulation, and systemic lipid remodeling (11–15, 18). In high-fat diet-induced obesity, MR has been reported to induce hepatic FGF21 expression, activate AMPK/PGC-1α signaling, restore lipid and bile acid metabolism, and improve metabolic homeostasis (15). These findings suggest that MRD may provide a nutritional strategy for improving obesity-associated metabolic disorders, potentially through coordinated regulation of methionine-related methyl-donor metabolism, hepatic lipid remodeling, and inflammatory responses. The most prominent metabolic benefit is the coordinated increase in both energy intake and expenditure. This increase is more pronounced in the case of energy expenditure (20, 21).

We hypothesized that MRD may modulate methionine-related methyl-donor metabolism and SAM-dependent phospholipid methylation, particularly in the hepatic PEMT pathway, resulting to the modulation of SAM. We utilized metabolomics, lipidomics, serum biochemical analyses, ELISA for inflammatory factors, and Listeria *monocytogenes infection* models, to systematically elucidate the regulatory effects of MRD on lipid metabolism and associated physiological functions during weight loss. This study was aimed to explore the effect of MRD restriction on the physiological effects of HFD-induced obese mice like hepatic lipid remodeling, inflammatory responses, and antibacterial defense. The findings are expected to provide a theoretical foundation and inform practical strategies for the management of obesity-related metabolic diseases.

## 2 Materials and methods

### 2.1 Materials

All reagents used for HPLC analysis were HPLC grade, methanol (A456-4, Thermo Fisher), acetonitrile (AC610010040, Sigma), chloroform (650498, Sigma). SETA (NSK-A, Cambridge Isotope Laboratories). Lipidomix, PC (17:0), PE (17:0), and PS (14:0) were obtained from Avanti. All other reagents used were analytical grade.

### 2.2 Animals

Wild-type C57BL/6 mice were purchased from GemPharmatech Co., Ltd. (Nanjing, Jiangsu, China). All mice were housed in pathogen-free conditions in a temperature-controlled environment at 22–24°C with a 12-h light/dark cycle. All animal experiments were conducted with the approval of the Animal Care and Use Committee of Zhejiang University, and the number of project license was ZJU2024026.

After one week of acclimatization, 18 mice aged 6–8 weeks were switched to a high fat diet (60 kcal% fat, D12492, Research Diets, Inc.) and maintained on this diet for 16 weeks, with three mice per cage. Mouse body weight was monitored weekly. Starting from week 16, the mice were divided into three groups and switched to different diets: the high-fat diet (HFD) group, the normal methionine control (NC) group (0.86% methionine, M20121704, Shanghai Biopike Biotechnology Co., Ltd.), and the methionine-restricted (MRD) group (0.17% methionine, M22121202, Shanghai Biopike Biotechnology Co., Ltd.). The experimental period lasted for 21 days, which was selected according to pre-experiment and report by Fang et al. (19). During the 21-day dietary intervention period, all of mice were housed individually, with one mouse per cage, to enable accurate monitoring of food intake, water consumption, and body weight. Food and water were provided *ad libitum* and measured daily at the same time. Daily food intake was calculated as the difference between the amount of food provided and the remaining food. Daily water intake was calculated as the difference between the initial and remaining water volume. Body weight was recorded daily throughout the intervention period. At the end of the 21-day dietary intervention, mice were fasted for 4 h before euthanasia to standardize the metabolic state before sampling, reducing the interference of acute post-meal metabolic fluctuations caused by recent food intake on serum biochemistry, lipidomics and metabolomics analyses. Blood and liver tissues were then collected, rapidly frozen in liquid nitrogen, and stored at −80°C until further analysis.

### 2.3 Biochemical analysis of serum

Total cholesterol (TC), triglyceride (TG), high-density lipoprotein cholesterol (HDL-C), and low-density lipoprotein cholesterol (LDL-C), glutamic pyruvic transaminase (ALT), glutamic oxaloacetic transaminase (AST) in serum were determined using commercial kits (Nanjing Jiancheng Bioengineering Research Institute Co., Ltd., Nanjing, China).

### 2.4 Enzyme-linked immunosorbent assay (ELISA)

Serum was collected during the experiments, and the levels of IL-6, IL-1β and TNF-α were measured using ELISA kits (NeoBioscience Technology Co., Ltd.) according to the manufacturer's instructions.

### 2.5 *Listeria monocytogenes* infection experiments

Another 12 male C57BL/6 mice aged 6–8 weeks were used for the *Listeria monocytogenes* infection experiment. Briefly, all of mice were fed with a high-fat diet for 16 weeks to establish obesity model, and then were randomly assigned into NC and MRD groups (*n* = 6) for a 21-day dietary intervention. The experimental protocol was performed according to the method described in previous (22). The preserved *Listeria monocytogenes* strains were inoculated onto dry brain-heart infusion (BHI, Beijing solarbio science & technology co., ltd.) agar plates from the refrigerator at −80°C and cultured overnight at 37°C. Single colonies were selected and cultured overnight in 2.0 mL BHI liquid medium (37°C, 180 rpm). The overnight cultures were diluted in the same medium to an OD_600_ of 0.1 and shaken at 37 C until the OD_600_ reached between 0.6 and 0.8. Bacteria were collected by centrifuging (6,000 RPM, 10 min) for three times, washed with sterile PBS solution, and finally adjusted to an OD_600_ of 0.6 (1 × 109^ CFU/mL) with PBS solution.

Obesity mice were randomly divided into two groups: NC group and MRD group, bacterias was injected into the mice via the tail vein at a dose of 2 × 10^5^ CFU per mouse. After 48 hours, the mice were immediately euthanized to avoid introducing an additional metabolic stress that could potentially affect host immune responses or bacterial clearance, and the liver and spleen were collected under sterile conditions. Small pieces of liver and spleen tissue were weighed and placed in grinding tubes, to which, 10 x PBS (M/V) was added along with grinding steel balls. The mixture was homogenized and then diluted onto petri dishes containing BHI solid medium to detect colony formation. The entire process was conducted in a sterile environment to avoid contamination that could affect the experimental results.

### 2.6 Extraction and quantification of liver and serum metabolites

#### 2.6.1 Extraction of metabolites from liver tissue

Metabolites from liver tissue and serum were extracted following previously described methods (23). Liver tissue (5.0 mg) was cut on dry ice with a knife and transferred to an empty centrifuge tube. For each sample, 400 μL of 60% methanol and 400 μL glass beads were added, followed by homogenization using the automatic sample grinder (JXFSTPRP, Jingxin) at 67 Hz for 5 min (repeated 5 times). During bead beating, the sample was freeze-thawed 5 times with liquid nitrogen. A hole was punctured in the bottom of the screw-cap tube using a needle, which was then placed into a new EP tube. Centrifugation was performed at 4 C and 4,000 g for 2 min, followed by washing with 200 μL methanol and re-centrifugation. The supernatant (500 μL) was collected and centrifuged at 4 °C and 15,000 g for 15 min, 450 μL of the solution was transferred to a new tube and re-centrifuged. The solution was transferred to a new tube twice, then dried using a Labconco vacuum concentrator and stored at −80°C until analysis.

Serum (5 μL) was resuspended in 120 μL MS-grade 60% methanol with homogenized at 67 Hz for 30 s. The sample was placed on ice for 30 min, followed by centrifugation at 15,000 g for 15 min at 4 C. Then 110 μL of the solution was transferred to a new tube and recentrifuged. The upper phase was divided into 2 EP tubes (50 μL each), with no residue from the bottom included. Finally, the samples were dried using a vacuum concentrator (Labconco) and stored at −80°C until analysis.

#### 2.6.2 Detection of metabolites by LC-MS

For metabolite preparation, each polar dry pellet was redissolved in 120 μL of 60% acetonitrile (AC610010040, Sigma) with 1/50 volume of amino acid standards mix set A. Metabolites were quantitated by LC–MS/MS with a triple quadrupole mass spectrometer (QTRAP 6500 + System; AB SCIEX) using previously established methods (24). Briefly, the redissolved metabolites were separated chromatographically on a SeQuant Zic-hydrophilic interaction liquid chromatography (pHILIC) column (5 μm polymer 150 × 2.1 mm; MilliporeSigma) using a high-performance LC system (ExionLC AD System) coupled to a triple quadrupole mass spectrometer (QTRAP 6500 + System, AB SCIEX). For targeted metabolomics, we performed a 34 min LC on the pHILIC column at a flow rate of 0.15 mL/min, with 20 mM ammonium carbonate and 0.1% (vol/vol) ammonium hydroxide as solvent A and 100% acetonitrile as solvent B. The following gradient was used: 80% solvent B for 0.01 min, 20% solvent B for 20 min, 80% solvent B for 20.5 min and 80% solvent B for 34 min. Metabolites were detected by MRM transitions in positive and negative modes.

### 2.7 Extraction and quantification of liver lipids

#### 2.7.1 Extraction of lipid from liver tissue

Lipids from liver tissue were extracted using chloroform/methanol (2:1, v/v) following previously described methods (25). Briefly, 5 mg of liver tissue was collected, weighed, and resuspended in 0.5 ml LC-MS-grade methanol (A456-4, Thermo Fisher) for bead-beating lysis. The whole lysate and precipitate were transferred to a glass tube, and 2.5 ml of chloroform (650498, Sigma) along with 0.4 ml of 50 mM citric acid (A610055, Sangon) were added for phase separation. The bottom lipid phase was collected, dried using a vacuum concentrator (Labconco), and stored at −80°C until analysis.

#### 2.7.2 Extraction of lipid from serum

5.0 μL serum was resuspended and homogenized in 120 μL MS-grade 60% methanol. Chloroform, then citric acid plus chloroform were added with identical resuspension; centrifuged at 4,500 rpm for 1 min (RT). The upper phase was aspirated, and the bottom phase was divided into 2–3 tubes. Finally, lipid samples in the organic phase were dried using a vacuum concentrator system.

#### 2.7.3 Detection of lipids profiling by LC-MS

Dried lipid extracts were resuspended in LC-MS-grade isopropanol, acetonitrile, and water (2:1:1) with 17:0 PC, 17:0 PE, and 14:0 PS spike-in standards from Lipidomix. The redissolved lipid samples were quantitated using LC–MS/MS with a triple quadrupole mass spectrometer (QTRAP 5500+System; AB SCIEX). Lipid separation was achieved on a C18 column (ACQUITY UPLC BEH C18, 130 Å, 1.7 μm, 2.1 mm × 50 mm), followed by quantification through multiple reaction monitoring (MRM) in positive mode for neutral lipids like TAG, and in negative mode for phospholipids. LC configuration: Buffer A: 33.3% acetonitrile, 33.3% methanol, 33.4% water, and 5 mM ammonium acetate; Buffer B: 100% isopropanol containing 5 mM ammonium acetate. The flow rate was 0.15 mL/min using the following method: 0 min, 20% buffer B; 1 min, 20% buffer B; 3 min, 60% buffer B; 13 min, 98% buffer B; 13.1 min, 20% buffer B; 16 min, 20% buffer B. Retention times for each MRM peak were compared. Quantification of peak areas was then performed using Analyst Software OS v2.0.

### 2.8 Statistical analysis

Sample size estimation was performed using G^*^Power software, version 3.1.9.7 (26). The expected effect size was estimated based on our preliminary data and previous mouse studies using methionine-restricted dietary interventions (23). The figures was obtained by GraphPad Pris 9 and Excel softwares. Data are presented as mean ± SD. An unpaired two-sided Student's *t* test was used to assess significance between two groups. Statistical significance of three groups was determined by one-way ANOVA with Tukey's multiple comparisons test. ^*^p < 0.05 and ^**^p < 0.01 represented the significant different of samples.

## 3 Results

### 3.1 Effects of MRD and NC diets on body weight, tissue mass, and serum biochemical parameters in HFD-induced obese mice

**Figure 1:**
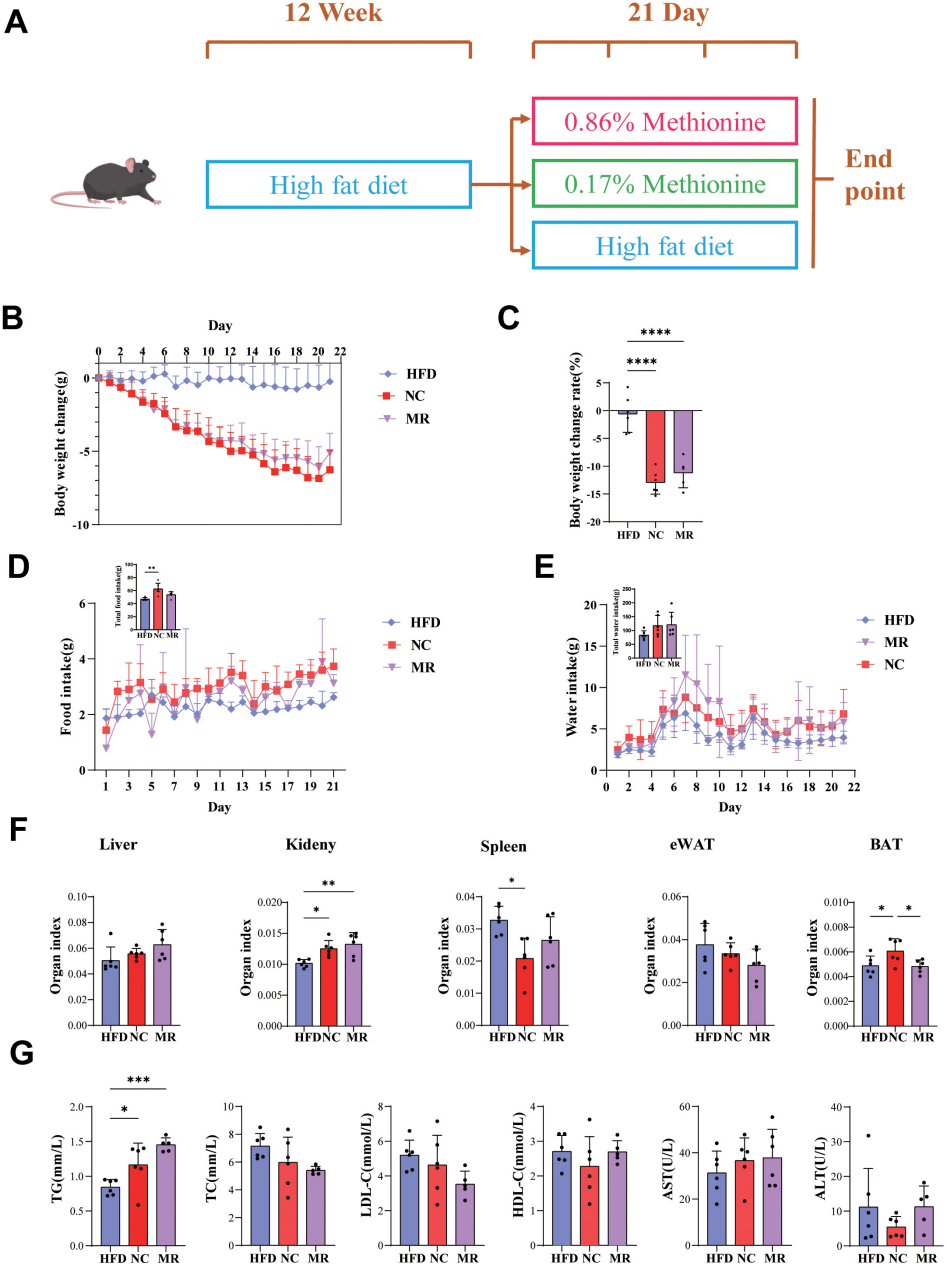
Methionine restriction in normal diet modulates body weight and tissue mass in HFD-induced obese mice relative to normal-methionine diet. **(A)** Schematic of the experimental design; **(B, C)** The effects of NC and MRD diet on the body weight of obese mice. **(D, E)** The effects of NC and MRD diet on the food intake and water intake of obese mice. **(F)** The effects of NC and MRD diets on the tissue weights of obese mice. **(G)** The effects of NC and MRD diets on the serum biochemistry of obese mice (*n* = 5~6). The symbols indicate statistical significance: ^*^p < 0.05, ^**^p < 0.01, ^***^p < 0.001, and ^****^p < 0.0001.

The biological effects of methionine as an essential amino acid have been extensively studied in the context of metabolic diseases. High-fat-induced obese mice were divided into three groups, HFD, NC and MRD groups, fed different diets and subjected to dietary interventions for 21 days, and the experimental flow chart is shown in Figure 1A. It was found that NC and MRD diets all resulted in a significant reduction in body weight of the obese mice, they were reduced by 12.9% and 11.2%, respectively (Figures 1B, C), whereas there was no significant change in the amount of water consumption. The total food intake of the NC group was significantly higher than that of the HFD group. From the daily food intake data, it can be seen that after 17 days, the average daily food intake of the NC group and the MRD group was higher than that of the HFD group (Figures 1D, E, *p* > 0.05), which suggests that the weight reduction was caused by a reduction in dietary energy intake and was not affected by dietary methionine content. Replacing a high-fat diet with a normal diet is a common strategy for healthy fat loss. Because a normal diet is less energy-dense and is often rich in dietary fiber, which improves satiety and thereby reduces total energy intake (27). Tissue indices were calculated by normalizing tissue weight to final body weight. No significant differences in liver index were observed among the three groups. Compared with the HFD group, kidney index was significantly increased in both the NC and MRD groups, which may partly reflect the reduction in final body weight after dietary switching rather than an absolute increase in kidney mass. The spleen index was significantly lower in the NC group than in the HFD group. There was no significant difference in the eWAT index among the three groups. In addition, the BAT index was significantly increased in the NC group compared with both the HFD and MRD groups, suggesting relative preservation or remodeling of BAT after switching to the normal control diet (Figure 1F). After 21 days of dietary intervention, mice in the NC and MRD groups (*n* = 5) exhibited significantly higher serum TG levels compared to the HFD group (Figure 1G). However, there were no significant changes in TC, LDL, HDL, and liver inflammation markers such as ALT and AST (Figure 1G). Furthermore, no differences were observed in any of these indices between the NC and MRD groups. This suggested that the observed phenomenon could be attributed to the transition from a high-fat diet to a balanced diet rather than to the differing methionine content in the diets.

### 3.2 MRD does not alter serum and hepatic metabolism in obese mice after weight loss

Metabolites in serum can reflect the state of systemic metabolism, making it an important tool for studying disease mechanisms and biomarkers (28). In parallel, the liver serves as the central hub for regulating metabolic homeostasis, maintaining immune function, and controlling lipid balance in the human body (29).

**Figure 2:**
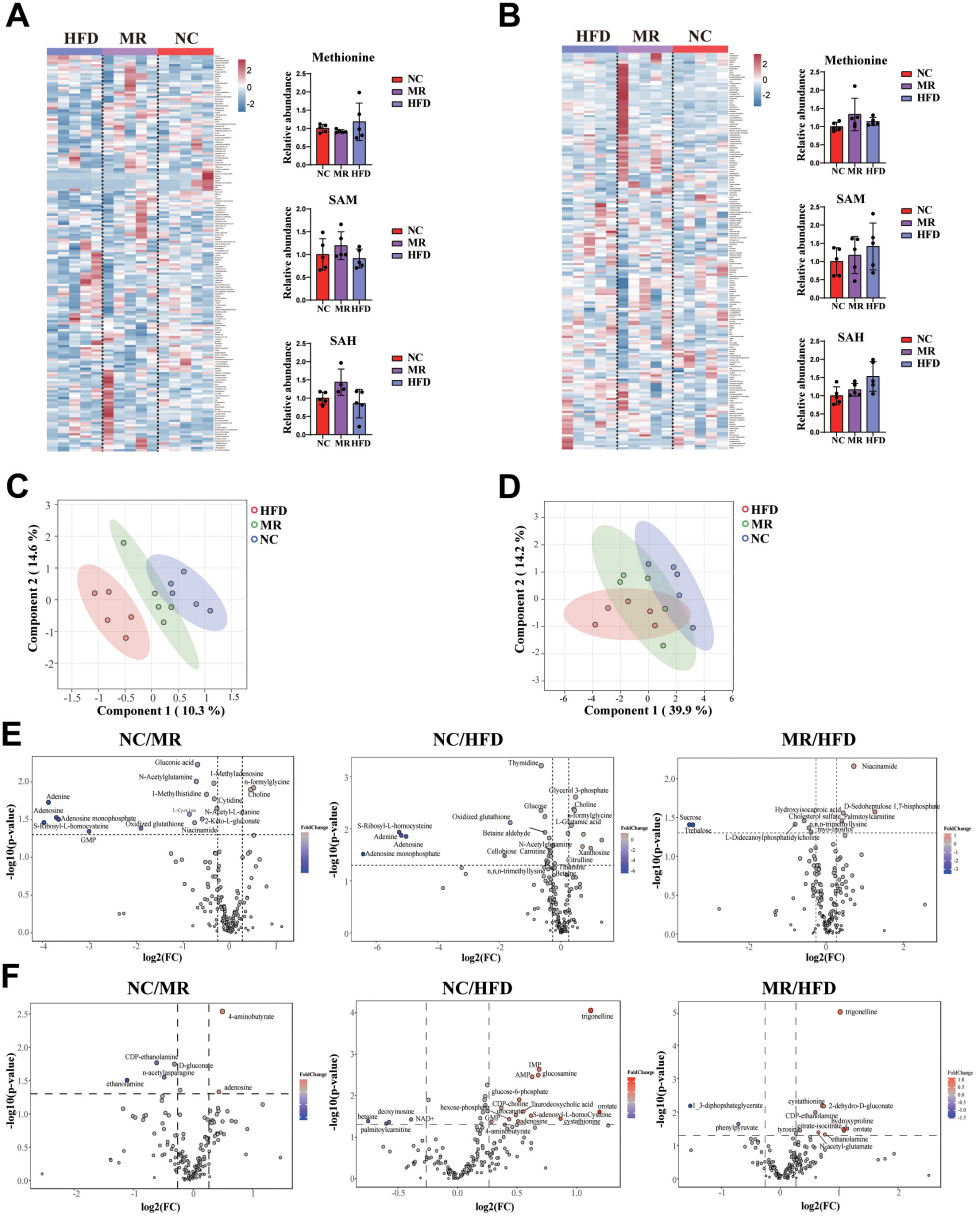
Comparative analysis of targeted serum and liver metabolomes in obese mice: impact of MRD vs. NC diets. Heatmap depicting abundances of serum **(A)** and liver **(B)** metabolism. Principal component analysis was performed of liver **(C)** and serum **(D)** metabolism. The volcano plot shows the differentially expressed metabolites in the liver **(E)** and serum **(F)** of mice from different diet groups.

To further assess the effects of NC and MRD diets on obese mice, we performed targeted metabolomics on serum and liver tissues of mice using LC-MS. To obtain an overview of systemic and hepatic metabolic changes after dietary intervention, we first visualized the targeted metabolomic profiles of serum and liver using heatmaps (Figures 2A, B) and PCA (Figures 2C, D). We therefore further focused on key metabolites related to methionine metabolism and methyl-donor balance, including methionine, S-adenosylmethionine (SAM), and S-adenosylhomocysteine (SAH). No significant changes in methionine, SAM, or SAH were observed between the NC and MRD groups in either serum or liver, suggesting that short-term MRD did not markedly disrupt the total pool size of these methionine-cycle metabolites under the present experimental conditions. The liver metabolism results there were 22 and 19 differential metabolites in the NC and MRD groups compared with the HFD group, respectively, and 6 differential metabolites between the NC and MRD groups, include ethanolamine and CDP-ethanolamine, adenosine, 4-aminobutyrate, D-gluconate, n-acetylasparagine (Figure 2F). The serum metabolism results showed there are 13 and 11 differential metabolites in the NC and MRD groups compared with the HFD group, respectively, and 17 differential metabolites between the NC and MRD groups were detected. The differential serum metabolites mainly included gluconic acid, 1-methyladenosine, choline, cytidine, adenine, L-cystine, adenosine, adenosine monophosphate, S-ribosyl-L-homocysteine, niacinamide, oxidized glutathione, and GMP etc. These metabolites were mainly associated with purine/nucleotide metabolism, methionine-cycle and one-carbon metabolism, glutathione-related redox metabolism, amino acid derivative metabolism, and choline/phospholipid precursor metabolism (Figure 2E).

### 3.3 MRD can specifically induce lipid remodeling during weight loss in obese mice

**Figure 3:**
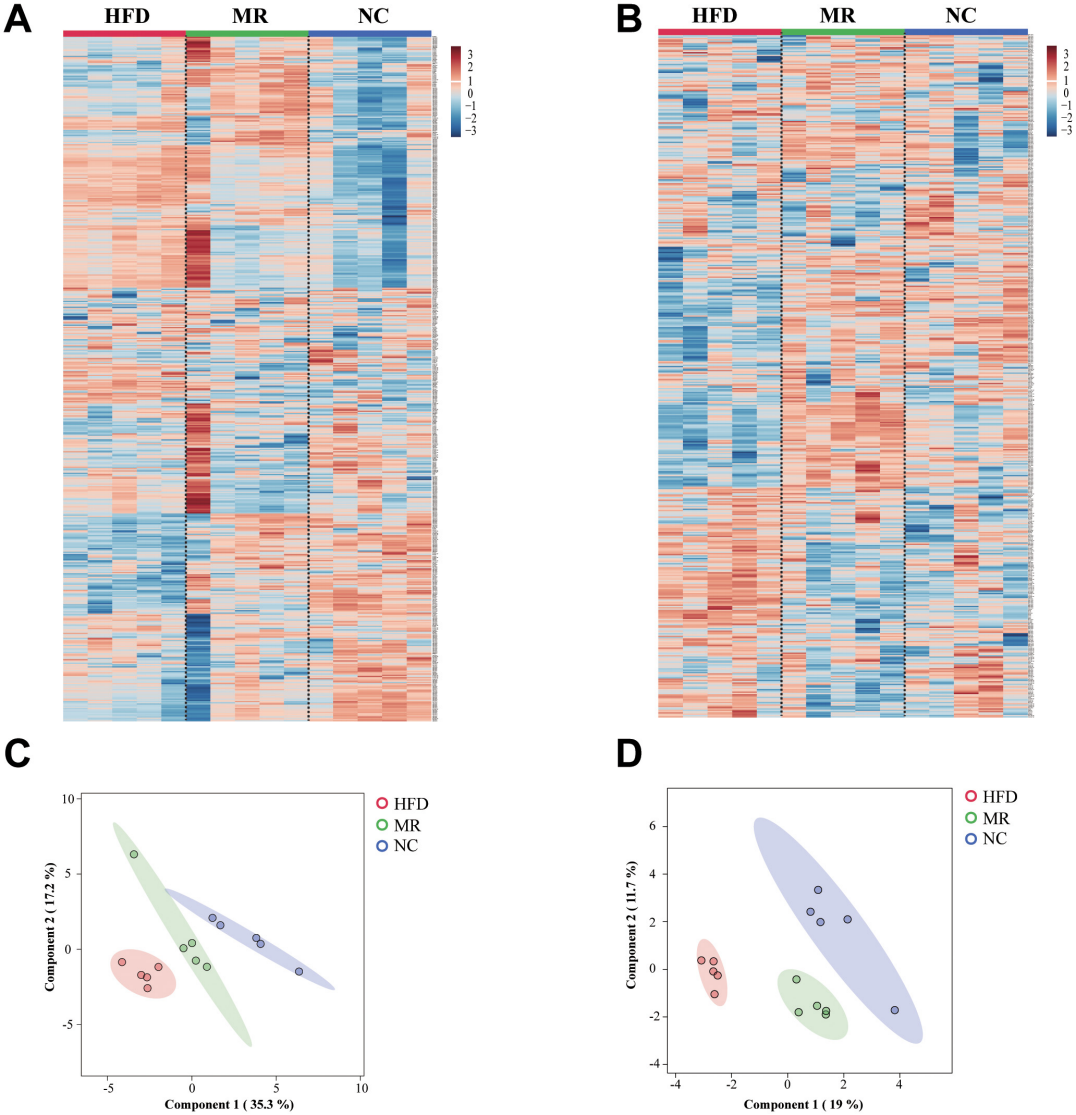
Effects on global liver/serum lipidomes in mice. Heatmap depicting abundances of liver **(A)** and serum **(B)** lipid. Principal component analysis was performed of liver **(C)** and serum **(D)** lipid.

Lipids perform and regulate many critical functions, including redox homeostasis, energy storage, intracellular and extracellular signaling, induction and resolution of acute and chronic inflammation, and maintenance of electrochemical gradients in subcellular intercellular compartments. To investigate the effects of NC and MRD diets on the liver and serum lipidomics of high-fat-induced obese mice, the lipidomes of liver and serum were analyzed by LC-MS. As shown in Figure 3, the identified lipids belonging to 12 subclasses were divided into two major groups of neutral lipids and phospholipids, with a total of 605 species in the liver (Figure 3A) and 557 species in the serum (Figure 3B). As shown in the PLS-DA score plot (Figures 3C, D), a clear spatial separation of NC, MRD, and HFD were observed, indicating that the lipidomics of mice after dietary intervention showed different responses to normal dietary lipid reduction with different methionine contents.

**Figure 4:**
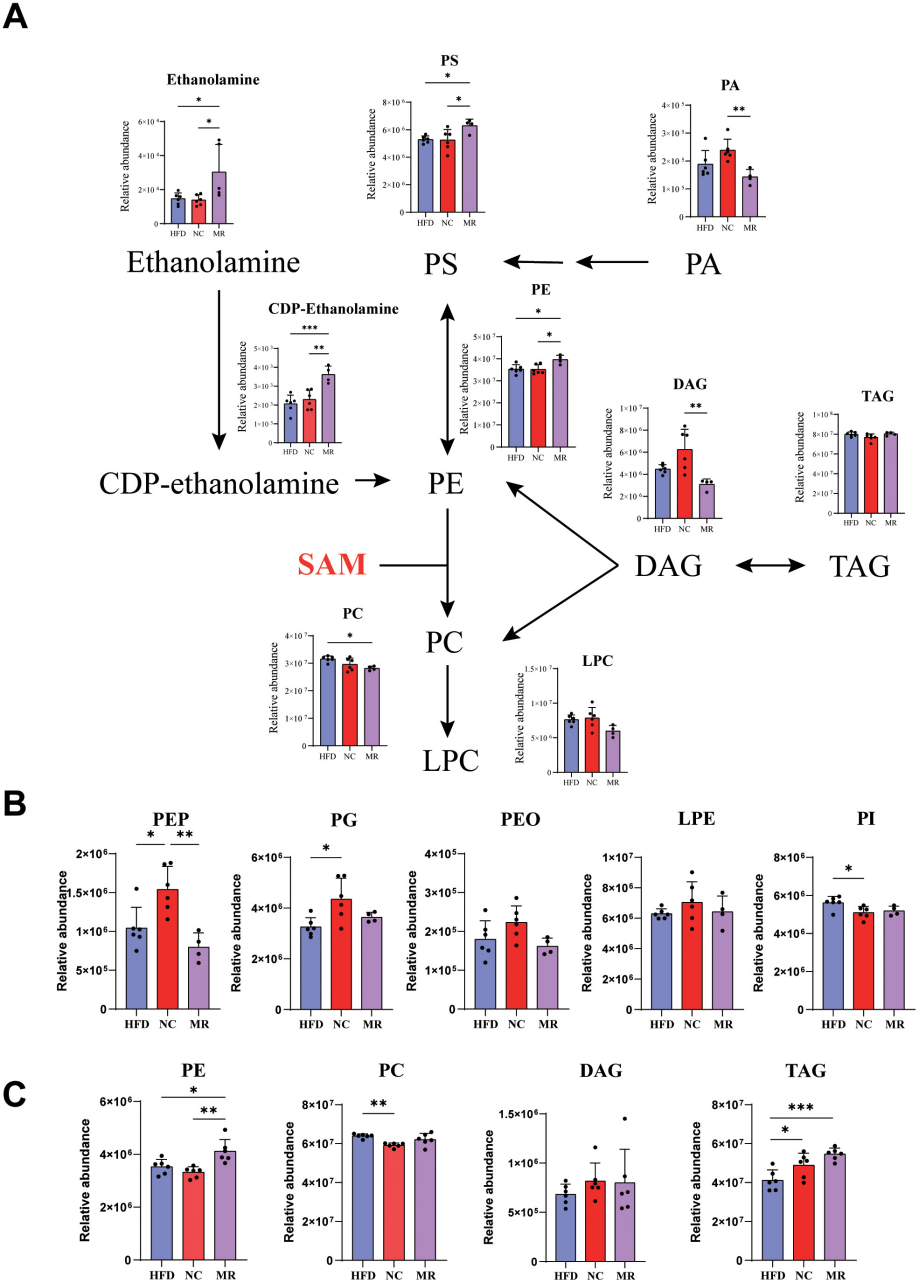
MR diet alters levels of key phospholipids and neutral lipids in liver and serum of HFD-induced obese mice. **(A)** The MRD regulates liver lipid metabolism in mice by altering PE. **(B)** Lipids that are insensitive to the regulation of lipid metabolism by the MRD. **(C)** The levels of PE, PC, TAG, and DAG in the serum. The symbols indicate statistical significance: ^*^p < 0.05, ^**^p < 0.01, ^***^p < 0.001.

Further fractionation of liver lipidomics revealed that the PE and PS in MRD group were significantly elevated and PA, LPC, and DAG were reduced as compared to the NC group, but there was no significant change in PC (Figure 4A). These results suggest that MRD selectively remodeled hepatic phospholipid metabolism. Although PEMT uses SAM to catalyze the conversion of PE-to-PC, targeted metabolomics showed no significant reduction in methionine or SAM levels in the MRD group, targeted metabolomics mainly reflect the steady-state pool size of metabolites, whereas metabolic flux reflects the turnover rate of metabolite through specific pathways. Thus, the observed phospholipid remodeling is not to be explained by simple depletion of methionine or SAM pools, because it may reflect altered metabolic flux, methylation demand, or lipid-metabolic adaptation (30, 31). The unchanged PC level suggestted that hepatic PC homeostasis was largely maintained, whereas the increases in ethanolamine and CDP-ethanolamine levels indicated the alteration of PE-related Kennedy pathway metabolism. Together with increased PS and decreased PA and DAG levels, these findings suggest that MRD intervention may shift lipid allocation among hepatic glycerophospholipid metabolic pathways. In addition, we similarly found the accumulation of PE in serum lipids (Figure 4C), but the other lipids did not show the same changes (Figure 4B). This may be that different tissues and organs in mice respond differently to MRD, and blood is the material exchange center of the organism, leading to the difference between the serum lipid group and the liver lipid group (32, 33).

**Figure 5:**
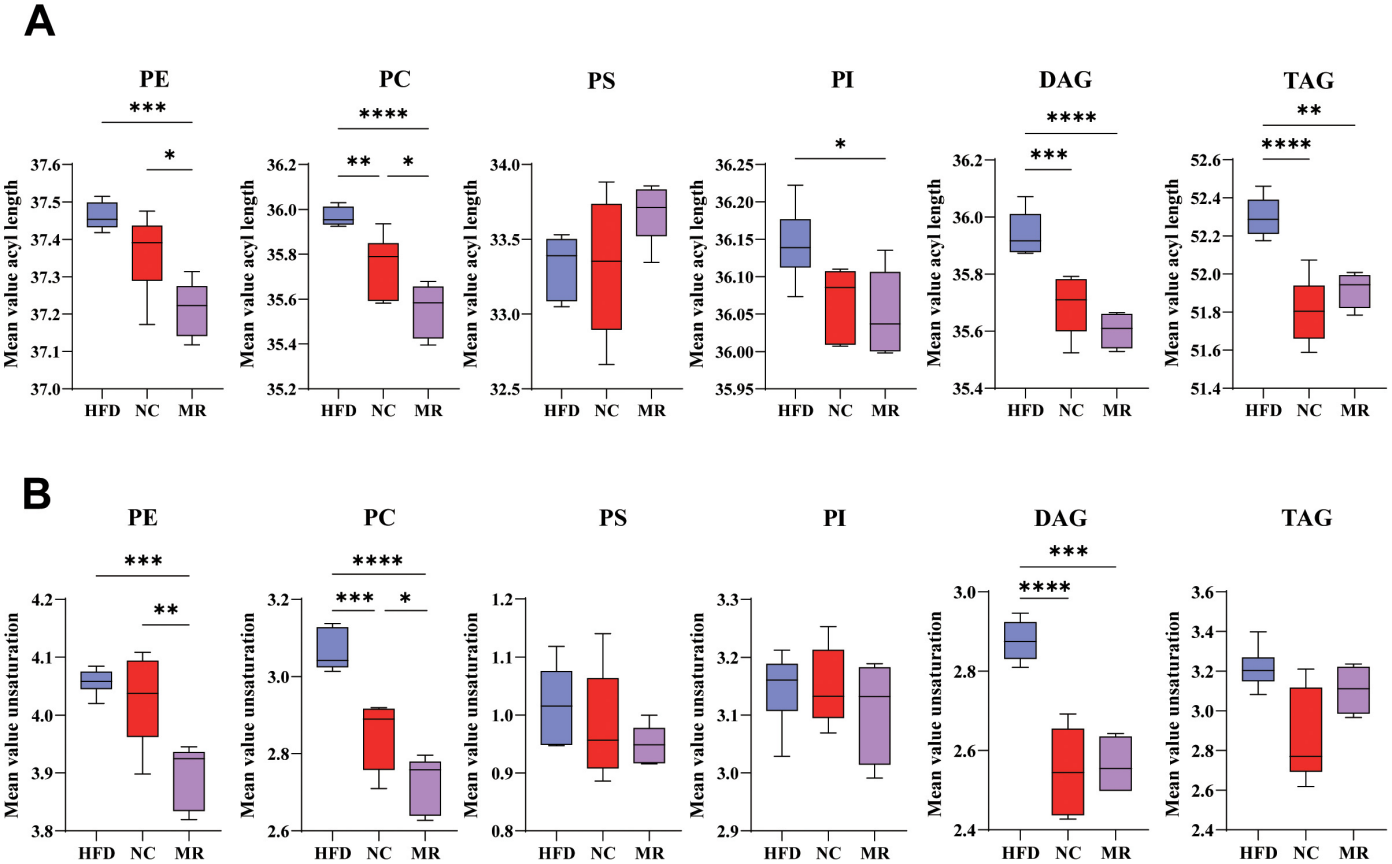
**(A)** Mean chain length (number of carbons) and **(B)** unsaturation (number of double bonds per fatty acid) of major lipid classes in liver lipid. The symbols indicate statistical significance: ^*^p < 0.05, ^**^p < 0.01, ^***^p < 0.001, and ^****^p < 0.0001.

Phospholipids are the main components of cell membrane structure, and their saturation and acyl chain length affect the fluidity of the cell membrane to maintain normal cellular function (34). We analyzed the lipidomics data more deeply in terms of the chain length of fatty acid acyl chains (Figure 5A) and the saturation of fatty acid chains (Figure 5B) for the two neutral lipids DAG and TAG, and the four phospholipids PE, PC, PI, and PS, which were the most abundant phospholipids in cells. The results showed that, compared to the HFD group, the average chain lengths of DAG, PE, and PC, as well as the PC and DAG saturation were significantly decreased in both the NC and MRD groups. What's more, the PE and PC level in MRD group were significantly lower in chain length and saturation than NC group.

We found that MRD induces accumulation of PE and PS, this specific lipid remodeling can alter the composition and structure of cell membranes and various organelle membranes, thereby influencing the physicochemical properties and functional of the membranes. Ultimately, it continuously affects the physiological homeostasis and health status of the body by regulating the overall function of the cells. (8, 35–37). Therefore, we examined serum inflammatory factors and resistance to bacterial infection in mice following MRD dietary intervention to further validate MRD's effects on the mouse organism.

### 3.4 MRD reduces inflammation levels in obese mice after weight loss and enhances resistance to Listeria infection

**Figure 6:**
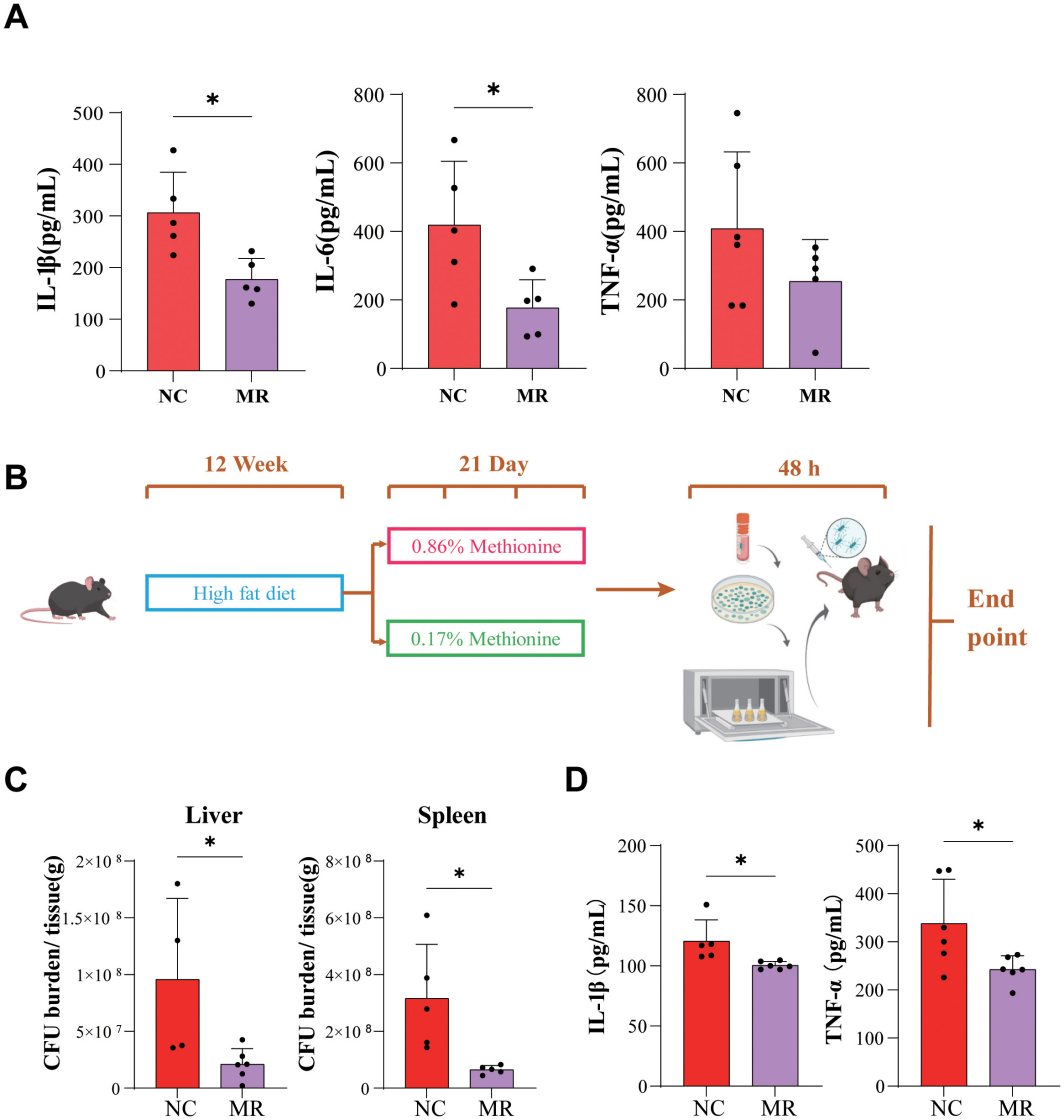
Effects of MRD interventions on inflammatory cytokine levels and bacterial infection resistance in obese mice. **(A)** The effect of different diet on inflammatory factrs in serum (*n* = 5~6). **(B)** Schematic of the bacterial infection experimental design. **(C)** Effect of MRD on the colonization ability of bacteria in liver and spleen (*n* = 4~6). **(D)** Effect of MRD on inflammatory factors in serum after bacterial infection (*n* = 5~6). The symbols indicate statistical significance: ^*^p < 0.05.

Obesity can lead to lipid disorders and chronic inflammation, it is one of the main causes of health problems in the organism. After the intervention of NC and MRD diets, significant changes in lipid composition were characterized in liver, and the IL-1β (*P* ≤ 0.05) and IL-6 (*P* ≤ 0.05) levels were significantly lower in the MRD group than in the NC group. In contrast, TNF-α was significantly higher in the HFD group than in both the NC (*P* ≤ 0.05) and MRD groups (*P* ≤ 0.01), whereas no significant difference was observed between the NC and MRD groups (Figure 6A). These findings indicate that MRD selectively reduced IL-1β and IL-6 compared with the normal methionine control diet, while dietary switching from HFD reduced circulating TNF-α levels, To verify this result, we constructed a bacterial infection model by injecting Listeria monocytogenes to mice that had undergone 21 days of dietary intervention (Figure 6B). The livers and spleens were removed from the abdominal cavities of the mice by aseptic technique after 48 h of infection, following by homogenization and dilution, monoclonal clones on the plates were counted on alternate days. It was found that the liver and spleen of mice in the MRD group had significantly lower bacterial loads than those in the NC group (Figure 6C). The pro-inflammatory factors IL-1β and TNF-α in serum were also significantly lower (Figure 6D). This suggests that during weight loss, a low-methionine normal diet increases the ability of mice to resist the risk of bacterial infections and improves body immunity compared with NC.

## 4 Discussion

The exploration of various weight-loss strategies, such as extreme calorie restriction and pharmacological interventions, often come with potential bodily harm, making it challenging to strike an ideal balance between “weight loss” and “health (38). The MRD, as an emerging metabolic intervention, has been demonstrated to improve insulin resistance and reduce fat accumulation in obese mice. However, the specific mechanisms by which it synergistically regulates hepatic lipid metabolism in obesity models remain unclear (15). This study employed a high-fat diet-induced obese mouse model and conducted a 21-day dietary intervention using two normal feeds with differing methionine contents. By integrating liver/serum metabolomics, liver lipidomics, and serum physiological biochemical indicators, the metabolic regulatory effects of MRD diets were systematically evaluated. At the physiological level, both normal methionine feeds significantly reduced body weight, and this effect was independent of changes in food intake. Notably, compared to the continuous high-fat diet group, the TG levels significantly increased in mice after intervention, with the specific mechanism requiring further investigation.

Metabolomic analysis revealed only minor differences in metabolites between the MRD and NC group in both liver and serum, MRD did not significantly disrupt the overall metabolic network, as evidenced by limited changes in serum and liver metabolomes. The limited metabolomic response may be partly explained by the lower growth-associated methionine requirement and greater metabolic buffering capacity of adult mice. (7, 39, 40) Compared with young or rapidly growing mice, adult mice require less methionine for body growth, organ development, and tissue protein accretion. In mature animals, methionine utilization is mainly related to maintenance metabolism, protein turnover, redox balance, and methyl-donor homeostasis (39). Therefore, short-term MRD may be buffered by compensatory mechanisms such as nutrient recycling, homocysteine remethylation, and adaptive metabolic regulation, resulting in limited changes in the overall serum and liver metabolomes (41). Subtle shifts in metabolome data reveal that the MRD diet not only improves metabolic dysregulation in obesity, but also possesses protective properties that maintain metabolic homeostasis. Previous studies indicate that MRD primarily influences key intermediates in methionine metabolism pathways (such as homocysteine and SAM), while exerting minimal effects on global metabolic networks involving carbohydrates and amino acids (42, 43). This finding is highly consistent with the metabolomics results found in this study. From a physiological perspective, MRD's stabilizing effect on overall metabolism helps prevent exacerbation of lipotoxicity or inflammatory responses caused by extreme metabolic fluctuations (44, 45).

However, liver lipidomics results revealed that MRD exerts a distinct directional regulatory effect. Compared with the NC group, the MRD group exhibited significant accumulation of PE, PS in liver tissues, while PA, and DAG levels were markedly reduced. In our study, MRD did not significantly reduce the levels of methionine or SAM in the liver, indicating that the observed phospholipid rearrangement cannot be simply attributed to the depletion of the methionine or SAM pools (30). At the same time, the stable contents of methionine and SAM reflect the balance among dietary supply, endogenous regeneration, cellular utilization, and compensatory adaptation, which do not directly represent the flux of the pathway (30, 46). Even when the levels of methionine or SAM remain unchanged, there may still be changes in the turnover of the methionine cycle, alterations in the demand for methylation dependent on SAM, or changes in the flux of phospholipid methylation mediated by PEMT (47). Therefore, the phospholipid rearrangement in the liver caused by MRD may be more closely related to adaptive metabolic redistribution and transcriptional regulation of lipid metabolism rather than simply being associated with simple substrate deficiency.Concurrently, increase in PE levels may further downregulate the synthesis pathways of PA and DAG, resulting in a distinct lipid profile. Regarding inflammation and immunity, 21 days of MRD intervention significantly reduced the levels of serum proinflammatory factors IL-1β, TNF-α, and IL-6. Further bacterial infection experiments demonstrated that the MRD group exhibited significantly weakened bacterial colonization capacity in both liver and spleen tissues. This indicates that through the MRD diet, by altering the composition of cellular lipids—particularly those in cell membranes and organelle membranes, enhances the body's immune defense capabilities and improves resistance to infection.

Phospholipids serve as core structural components of cell membranes, particularly mitochondrial membranes and plasma membranes. Changes in their composition directly impact membrane stability and cellular function. PE, the second most abundant phospholipid in mammals, plays a crucial role in maintaining membrane protein conformation and structural integrity (48). In this study, MRD-induced accumulation of PE and PS, coupled with the suppression of pro-inflammatory factor release, collectively form the structural basis for improving metabolic disorders and susceptibility to infection. Therefore, we propose that the physiological regulation of obese mice by the MRD diet constitutes a multi-level, synergistic network process: Initiated by the remodeling of hepatic lipid metabolism. It ultimately achieves resistance to bacterial colonization through inflammation suppression.

These lipid alterations may provide a structural basis for the reduced inflammatory responses and enhanced antibacterial defense. PE and PS are important membrane phospholipids, and changes in PE/PC acyl-chain length and saturation can affect membrane fluidity, curvature, and membrane protein organization (49–53). Such changes may help maintain mitochondrial membrane integrity and reduce mitochondrial stress, ROS production, and mtDNA release, thereby limiting inflammasome activation and IL-1β secretion (49, 50). In addition, changes in plasma membrane lipid composition may influence host membrane organization and lipid raft-dependent interactions involved in Listeria monocytogenes entry or intracellular spread (54, 55). However, these mechanisms remain speculative because membrane fluidity, mitochondrial function, and bacterial invasion were not directly measured in this study.

This study has several limitations. The present study did not include normal diet-fed mice from the beginning, with or without methionine restriction; therefore, the findings mainly reflect MRD effects in established HFD-induced obesity rather than obesity prevention or responses under healthy metabolic conditions, the effect of methionine restriction on preventing the development of obesity and healthy animals will be investigated in our future study. In addition, only male mice were used, inguinal white adipose tissue was not investigated, and gut microbiome analysis was not performed, which limits the assessment of sex-specific, depot-specific, and microbiome-dependent effects. The effect of methionine-restricted diet on the gut microbiome and its metabolites of HFD mice need to be explaored in our future work. Finally, PEMT activity, methionine-cycle flux, and SAM-dependent phospholipid methylation flux were not directly measured; thus, the methionine–SAM–PEMT axis should be considered a potential mechanism requiring further validation.

## 5 Conclusion

In summary, short-term MRD during dietary weight-loss intervention was associated with selective remodeling of the hepatic lipid network, particularly changes in PE, PS, and PE/PC acyl-chain profiles. These lipid alterations may provide a structural basis for the observed changes in inflammatory responses and antibacterial defense. On one hand, the accumulated PE and PS play a crucial role in maintaining membrane potential, reducing reactive oxygen species production, this may be a key reason for the reduction in proinflammatory factors such as IL-1β, thereby alleviating chronic inflammation. On the other hand, alterations in the phospholipid composition of the cell membrane may physically hinder the efficiency of bacterial surface protein adhesion to host cells, resulting in potent anti-colonization capabilities. Overall, this study provides experimental evidence that MRD may serve as a nutritional intervention to modulate hepatic lipid remodeling and inflammatory responses during obesity management.

## Data Availability

The original contributions presented in the study are included in the article/supplementary material, further inquiries can be directed to the corresponding authors.
